# Comparison of the Abundance and Community Structure of N-Cycling Bacteria in Paddy Rhizosphere Soil under Different Rice Cultivation Patterns

**DOI:** 10.3390/ijms19123772

**Published:** 2018-11-27

**Authors:** Xiaomei Yi, Jing Yuan, Yuanhong Zhu, Xiaojian Yi, Qi Zhao, Kaikai Fang, Linkui Cao

**Affiliations:** 1 School of Agriculture and Biology, Shanghai Jiao Tong University, 800 Dongchuan Road, Shanghai 200240, China; yixiaomei@sjtu.edu.cn (X.Y.); zhaoq@sjtu.edu.cn (Q.Z.); fangkaikai@sjtu.edu.cn (K.F.); 2 Institute of Soil and Water Resources and Environmental Science, College of Environmental and Resource Sciences, Zhejiang University, Hangzhou 310058, China; yuanj1203@zju.edu.cn; 3 Shanghai Qingpu Modern Agricultural Park, 6800 Old Zhufeng Road, Shanghai 201715, China; guti14zdx@hotmail.com; 4 Shanghai Zizai Qingxi Agricultural Development Co., Ltd, 325 Zhengxing Road, Shanghai 201715, China; yixiaojian89@126.com

**Keywords:** rice-frog cultivation, rhizosphere soil, N-cycling, bacteria, 16S rRNA

## Abstract

Eco-agricultural systems aim to reduce the use of chemical fertilizers in order to improve sustainable production and maintain a healthy ecosystem. The aim of this study was to explore the effects of rice-frog farming on the bacterial community and N-cycling microbes in paddy rhizosphere soil. This experiment involved three rice cultivation patterns: Conventionally cultivated rice (CR), green rice-frog farming (GR), and organic rice-frog farming (OR). The rice yield, paddy soil enzyme activities, physicochemical variables and bacterial and N-cycling bacterial abundances were quantitatively analyzed. Rice-frog cultivations significantly increased soil protease, nitrate and reductase activity. Additionally, the *nir*S gene copy number and the relative abundance of denitrifying bacteria also increased, however urease activity and the relative abundance of nitrifying bacteria significantly decreased. The bacterial community richness and diversity of OR soil was significantly higher than that of the GR or CR soil. Nitrogen use efficiency (NUE) of GR was highest. The N-cycling bacterial community was positively correlated with the total carbon (TC), total nitrogren (TN) and carbon to nitrogen (C:N) ratio. The present work strengthens our current understanding of the soil bacterial community structure and its functions under rice-frog farming. The present work also provides certain theoretical support for the selection of rational rice cultivation patterns.

## 1. Introduction

Food security is becoming a global problem because of the increasing world population. Global agriculture is affected by the limited amount of land, the scarcity of water and climate change [1,2]. Rice is the main ingredient in the daily diets of about 3 billion people. Rice production is a key component in global food security. Moreover, more than 90% of the 163 million ha of rice cultivated worldwide is located in developing countries, especially in Asia [3]. In the past decades, rice production has increased because of the introduction of high-yielding varieties, increased mechanization and the large use of irrigation, pesticides and chemical fertilizers [4,5]. China is recognized as one of the most significant chemical nitrogen (N) fertilizer users and one of the largest rice producers in the world. Only 28.3% of the applied N is taken up by rice [6]. A significant proportion of the applied N is only temporarily retained in rice fields, i.e., substantial N is lost in nitrate leaching and runoff and in the emission of N oxides [7]. Thus, the management of N has become an important concern in high-yield rice systems [8]. 

In order to reduce nitrogen loss, the traditional agricultural system is under scrutiny again; traditional agricultural systems reflect a successful adaptation to different environments and are rich in biological diversity [9,10,11]. The recognition of the ecological legacy of these traditional agricultural systems and the integration of these unique conditions into future farm designs could help us to develop more sustainable agricultural practices. Ecological recycling agriculture (ERA) represents an organic agricultural system that aims to promote nutrient cycling efficiency [12,13]. Based on local and renewable resources, ERA efficiently integrates crop and animal production on an individual farm or on farms within a close proximity of one another. By increasing nutrient recycling, ERA may effectively enable a reduced amount fertilizer input into the agricultural system. Many studies have described the rice-fish, rice-duck, rice-turtle and rice-frog cultivation ecosystems and have shown how these practices reduced the rate of chemical fertilizer and pesticide application [11,14,15,16,17]. Paddy fields are important habitats of frogs [18]. It is well known that the frog feeds on crop pests. Their predatory behavior in paddy fields could reduce the incidence of rice pests, lowering the application of pesticides and contributing to biological control [19]. However, frogs have almost disappeared in paddy fields in many areas of China due to human malicious hunting and excessive pesticide application in the past few decades [20,21]. As frog populations are significantly reduced in paddy fields, rice pests become more and more rampant and grain yield largely decreases [22]. To deal with the infestation of pests, more pesticides have been applied to the fields, causing more severe soil and water pollution in agriculture, leading to a higher content of pesticide residues in rice. The rice-frog cultivation ecosystem, combined with organic farming techniques such as the application of organic fertilizers, represents an ecosystem with effective pest control, where there is less use of chemical fertilizers, decreased runoff loss, decreased leaching loss of N and good conservation of local soil and water resources [23]. Introducing commercial frogs in paddy fields could reduce the number of pests and lower pesticide application. However, most studies of this rice cropping pattern have focused on pests, disease and N loss control [18]; the mechanisms of a microorganism driven nitrogen cycle are rarely studied.

Rice plants typically are in close proximity to a highly abundant and diverse microbial community [24]. Soil microorganisms are important components of the paddy field ecosystem and key participants in the material cycling of paddy field ecosystems. Soil microbes participate in the energy flow and material cycling of the paddy field ecosystem by decomposing plant and animal residues and affecting the development of rice [25]. Microorganisms play fundamental roles in wetland biogeochemistry through their versatile functions [26]. Microbial communities respond to environmental alterations in a spatially and temporally highly dynamic fashion. Microbial activity, biomass and population dynamics could impact various chemical and physical conditions, strongly shaping wetland biomes and ecosystem functions [27]. N transformations in the soil are complicated because N can be present in different forms: Soluble and gaseous forms and as organic and inorganic compounds [28,29,30]. The abundances of functional genes are the best predictive variables of N transformation processes and cycling rates. Soil N dynamics and functional genes could be affected by different types of fertilization [31]. Studies on soil N cycling driven by microbes in ecological agriculture have been rarely reported globally, while the functional microorganisms of a rice-frog cultivar paddy have not been studied and assessed in detail. The purpose of this study is to explore the soil N cycling driven by microbes, aiming to provide theoretical guidance for the selection of rational rice cultivation patterns.

## 2. Results

### 2.1. Soil Enzyme Activities

**Figure 1:**
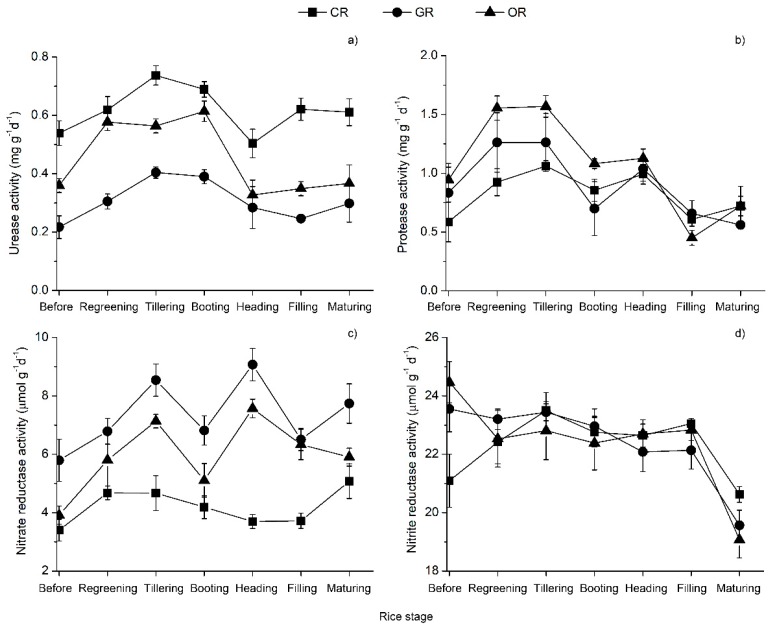
Variation in the soil enzyme activities in paddy rhizosphere soil. (**a**) Urease activity; (**b**) protease activity; (**c**) nitrate reductase activity; (**d**) nitrite reductase activity. The first sample was collected on June 14 (0 d) before base fertilization and the last sampling date was October 20. The seven stages are shown at the top. CR: Conventionally cultivated rice; GR: green rice-frog co-cultivation; OR: Organic rice-frog co-cultivation.

Rice-frog cultivation application impacted enzyme activities in soils and the effects on soil enzyme activities (urease activity, protease activity, nitrate reductase activity and nitrite reductase activity) are shown in Figure 1. The effects of rice-frog cultivation on urease activities were significantly different in CR, GR and OR soils. The urease activity in the three methods of growing rice increased before the tillering stage, after which it was inhibited. The urease activity in CR soil (0.504–0.737 mg g^−1^ d^−1^) was significantly higher than that in OR soil (0.327–0.614 mg g^−1^ d^−1^), which was significantly higher than that in GR soil (0.217–0.404 mg g^−1^ d^−1^), while protease activity decreased at the tillering stage, slightly increased at the booting stage and decreased after the heading stage. Although the soil protease activity of OR (0.451–1.645 mg g^−1^ d^−1^) was greater than that of CR (0.586–1.361 mg g^−1^ d^−1^) and GR (0.361–1.262 mg g^−1^ d^−1^), there were no significant differences exhibited among the three methods. There were marginally significant positive effects on the nitrate reductase activity in GR soil; the nitrate reductase activity ranged from 5.795 to 9.076 μmol g^−1^ d^−1^. The nitrate reductase activity in OR soil (3.914–7.568 μmol g^−1^ d^−1^) was significantly higher than that in CR soil (3.401–5.081μmol g^−1^ d^−1^). The nitrite reductase activity in the three methods showed no obvious changes during the entire growth period, however GR resulted in the highest cumulative N content of 1.35% in plants and the highest protein content of 9.72% (Table 1). In addition, the rice yield with GR was 8650.38 kg ha^−1^, which was similar with that of CR and significantly higher than that of OR. The rice yields with CR and OR were 8827.56 kg ha^−1^ and 7350.69 kg ha^−1^, respectively. The average yield with GR and CR was 8738.97 kg ha^−1^, representing an increase of 18.89% compared to OR.

### 2.2. Quantification of N-cycling Genes

**Figure 2:**
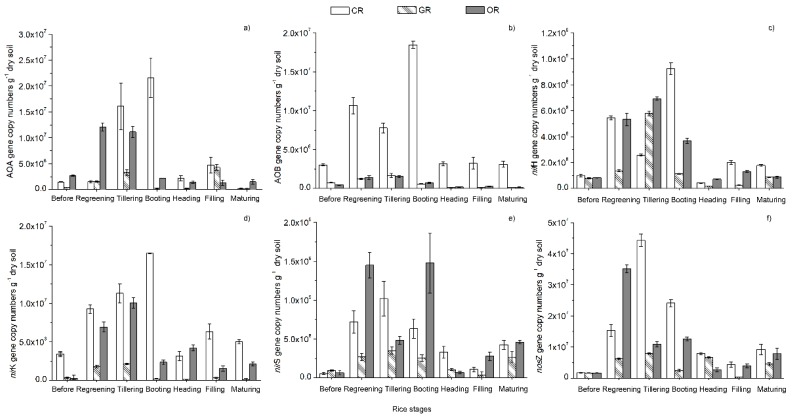
N-cycling gene copy numbers in paddy rhizosphere soil. (**a**) AOA gene copy numbers; (**b**) AOB gene copy numbers; (**c**) *nif*H gene copy numbers; (**d**) *nir*K gene copy numbers; (**e**) *nir*S gene copy numbers; (**f**) *nos*Z gene copy numbers. CR: Conventionally cultivated rice; GR: Green rice-frog co-cultivation; OR: Organic rice-frog co-cultivation.

The effects of the fertilizer treatments on the abundance of *nif*H, archaeal *amo*A (target AOA groups), bacterial *amo*A (target AOB groups), *nir*K, *nir*S, and *nos*Z genes in different growth stages of rice were determined using real-time PCR (Figure 2). All N-cycling gene copy numbers increased after transplantation and subsequently decreased until the filling stage. At the maturing stage, the N-cycling gene copy numbers decreased very significantly. From the regreening to maturing stage, the average *nif*H gene copy number was not significantly different among the three patterns. The AOB and *nir*K gene copy numbers varied significantly among the CR, GR and OR patterns (*p* < 0.05), with 7.31 × 10^6^, 6.48 × 10^5^, and 6.67 × 10^5^ copies g^−1^ dry soil and 7.82 × 10^6^, 7.24 × 10^5^, and 3.89 × 10^6^ copies g^−1^ dry soil, respectively. There were no significant differences in the AOA, *nir*S, and *nos*Z gene copy numbers between CR and OR, which were significantly higher than those of GR; the copy numbers were 6.50 × 10^6^, 1.43 × 10^6^, and 4.59× 10^6^ copies g^−1^ dry soil; 4.57 × 10^8^, 1.92 × 10^8^, and 5.97 × 10^8^ copies g^−1^ dry soil; and 1.52 × 10^7^, 4.22 × 10^6^, and 1.06 × 10^7^ copies g^−1^ dry soil, respectively. The ratios of AOA to AOB of CR, GR and OR were 0.08, 2.21 and 6.79, respectively.

Pearson analysis (Table 2) revealed that urease activity was positively correlated with the AOA, AOB, *nir*K, *nif*H, *nir*S, and *nos*Z gene copy numbers and the plant N content but negatively correlated with the grain protein contents. Protease activity was positively correlated with the NiRA and *nos*Z gene copy numbers and negatively correlated with rice yield. Correlations among AOA, AOB, *nir*K, *nif*H, *nir*S, and *nos*Z gene copy numbers were also significantly associated with each other except between AOB and *nir*S. The coefficient for the association of rice yield with AOB was 0.83. These findings indicated that the rice yield was significantly and actively related to the AOB gene copy number. There was a significant positive correlation between the plant N content and grain protein content.

### 2.3. Bacterial Community Structure in Paddy Rhizosphere Soil

A total of 332,990 high-quality sequences were obtained with a median read count per sample of 36,999. The high-quality reads were clustered using a >97% sequence identity into 192,580 microbial OTUs. Low-abundance OTUs (<5 total counts) were discarded. The Chao 1 and ACE indexes were calculated to estimate the community richness of each sample, and the Shannon and InvSimpson diversity indexes were used to evaluate the community diversity of each pattern. As shown in Table 3, the CR pattern when compared with the OR pattern shows the Chao1 and ACE richness indexes of the bacterial community were significantly increased, while the effect of the GR pattern was similar to that of CR. In terms of the diversity indexes, all three patterns resulted in a significantly different bacterial InvSimpson index, however the Shannon indexes of GR and CR were similar.

**Figure 3:**
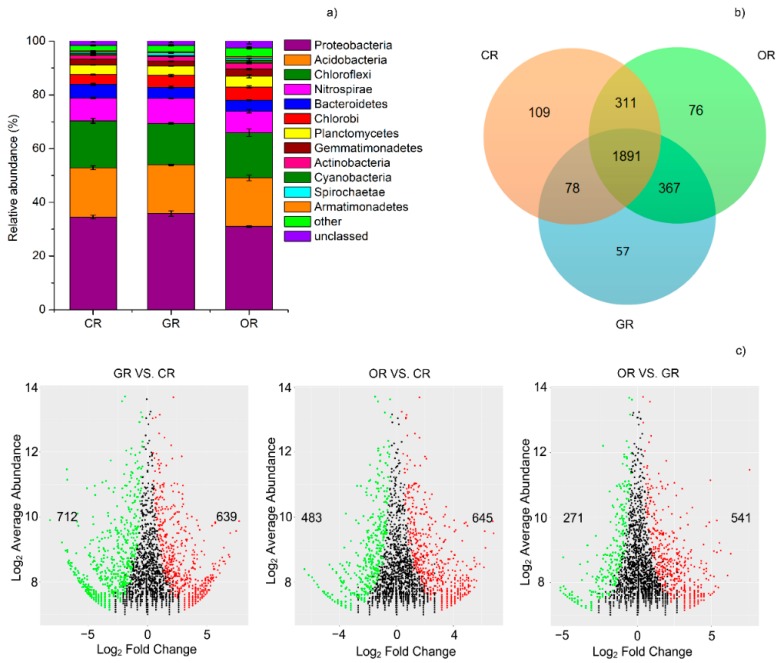
(**a**) Relative abundance of microbial populations at the phylum level in each paddy rhizosphere soil pattern. (**b**) Numbers of differentially enriched 16S rRNA operational taxonomic units (OTUs) in the paddy rhizosphere among the three patterns. (**c**) Enrichment and depletion of the OTUs for each pattern as determined by differential abundance analysis. Each point represents an individual OTU and the position along the y axis represents the abundance fold change compared with each pattern. CR: Conventionally cultivated rice; GR: Green rice-frog cultivation; OR: Organic rice-frog cultivation.

Through 16S rRNA sequencing, more than 12 bacterial phyla were identified: *Proteobacteria,* 33.79%; *Acidobacteria*, 18.18%; *Chloroflexi*, 16.60%; *Nitrospirae*, 8.57%; *Bacteroidetes*, 4.48%; *Chlorobi*, 4.30%; *Planctomycetes*, 3.72%; *Gemmatimonadetes*, 2.17%; *Actinobacteria*, 1.82%; *Cyanobacteria*, 0.70%; *Spirochaetae*, 0.78%; *Armatimonadetes*, 0.50%; and others, 2.55% (Figure 3a). The 16S rRNA sequencing indicated that *Proteobacteria* were the largest bacterial community. The CR (35.83%) and GR (34.52%) soil had a significantly greater proportion of *Proteobacteria* than the OR (31.01%) soil, whereas *Chloroflexi* was mostly depleted in the GR soil, OR soil contained a significantly greater proportion of *Gemmatimonadetes*, *Planctomycetes* and *Chlorobi* than CR or GR soil. The relative abundance of these phyla and the microbial diversity both decreased in the following order: OR, GR and CR. As shown in the Venn plot (Figure 3b), the OTUs enriched in the GR community were very successful in the rhizosphere soil, as 2336 out of the 2393 OTUs enriched in GR soil were also enriched in either the CR or OR communities or both. OR and GR shared the largest number of common OTUs (2258), while CR and GR shared the fewest (1969).

We concluded differential abundance analysis to distinguish OTUs that were significantly influenced by different rice cultivation patterns. Using OTUs from each pattern as a control and an adjusted P value cutoff of 0.1, “enriched OTUs (eOTUs)” and “depleted OTUs (dOTUs)” specifically represent OTUs that increased or decreased significantly (more than doubling or halving, respectively) in relative abundance in response to rice-frog cultivation. GR soil was most similar to OR soil, as indicated by the “tail” in the MA plot (Figure 3c), however an enrichment effect in GR soil is implied by the high ratio of statistically significant enriched OTUs compared with depleted OTUs in CR (712 vs. 639). In comparison, GR enriched many OTUs while simultaneously depleting a larger proportion of OTUs (483 vs. 645).

### 2.4. N-cycling Bacterial Community

**Figure 4:**
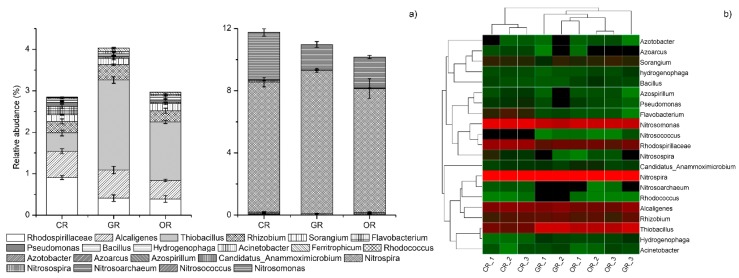
Relative abundances of N-cycling bacteria in the bacterial community and a heatmap at the genus level. CR: Conventionally cultivated rice; GR: Green rice-frog cultivation; OR: Organic rice-frog cultivation. Relative abundances (**a**) and heatmap (**b**) of N-cycling bacteria from the paddy rhizosphere at the genus level. The samples were grouped according to their similarity to one other. According to the clustering results, the samples are arranged horizontally. Similarly, the classification units also cluster according to their similarity to one other in different samples and were arranged vertically. Heatmap colors represent the relative abundance of OTUs (in percent). In (b), red represents a genus with a high abundance in the corresponding sample, and green represents a genus with low abundance.

At the genus level, 21 major N-cycling bacterial genera were identified (Figure 4a) and the cumulative abundances with the three patterns were GR > CR or OR. There were both nitrogen-fixing and denitrifying bacteria: *Thiobacillus* (1.10%), *Rhizobium* (0.60%), *Pseudomonas* (0.06%) and *Bacillus* (0.05%). *Thiobacillus* was the dominant genus, whose abundance in GR was significantly higher than that in OR and CR. *Azospirillum* (0.03%) *Azotobacter* (0.02%) and *Azoarcus* (0.02%) are nitrogen-fixing bacteria. For nitrifying bacteria, *Nitrospira* and *Nitrosomonas* were the predominant genera, with average relative abundances of 8.51% and 3.50%, respectively, across all patterns and relative abundances of CR > GR > OR. The relative abundances of denitrifying bacteria were 0.57% (*Rhodospirillaceae*), 0.54% (*Alcaligenes*), 0.20% (*Sorangium*), 0.11% (*Flavobacterium*), 0.04% (*Hydrogenophaga*), 0.04% (*Ferritrophicum*), 0.03% (*Acinetobacter*), 0.02% (*Rhodococcus*) and 0.03% (*Azospirillum*), and their cumulative abundances were 2.86% (CR), 4.04% (GR) and 2.97% (OR). From the heatmap (Figure 4b), the three patterns could be divided into RF (rice-frog cultivation, GR and OR) and conventionally cultivated rice (CR). There were two clusters of N-cycling bacterial genera. The first cluster consisted of *Azotobacter*, *Azoarcus, Sorangium*, *Hydrogenophaga*, *Bacillus*, *Azospirillum*, *Pseudomonas*, *Flavobacterium*, *Nitrosospira, Nitrosomonas*, *Rhodococcus* and *Nitrosococcus*, which preferred an environment with chemical N fertilization. Their relative abundances were lower in the RF group than in the CR group. The second cluster included *Candidatus Anammoximicrobium*, *Nitrospira*, *Nitrosoarchaeum*, *Rhodococcus*, *Alcaligenes*, *Rhizobium*, *Thiobacillus*, *Ferritrophicum* and *Acinetobacter*, and their relative abundances were increased by organic fertilization while decreased by chemical fertilization.

**Figure 5:**
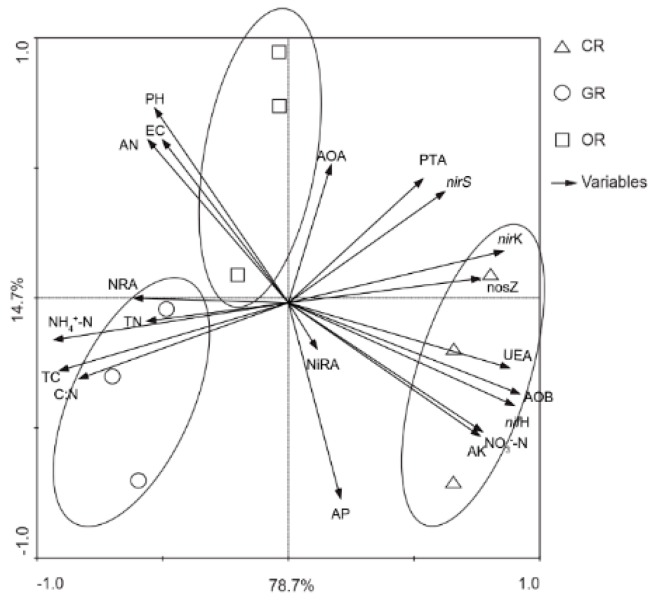
Redundancy analysis (RDA) of the associations between the N-cycling bacteria and soil variables in paddy rhizosphere soil. CR: Conventionally cultivated rice; GR: Green rice-frog co-cultivation; OR: Organic rice-frog co-cultivation. Environmental factors included are the soil pH; electrical conductivity (EC); total N (TN); total carbon (TC); nitrate N (NO_3_^−^-N); ammonium N (NH_4_^+^-N); available nitrogen (AN); available phosphorus (AP); available potassium (AK); carbon:N ratio (C: N); urease activity (UEA); protease activity (PTA); nitrate reductase activity (NRA); nitrite reductase activity (NiRA); and AOA, AOB, *nif*H, *nir*K, *nir*S, and *nos*Z gene abundances. Through 16S rRNA sequencing, we identified average OTUs for 21,396 species, 1064 of which belonged to N-cycling bacteria. Among them, species that were involved in cycling (see support material) and had average OTUs of ≥1 in the samples were selected for RDA.

The relationship between the variations in the community structure of the N-cycling bacterial community and environmental factors in paddy soils were assessed by redundancy analysis (RDA) (Figure 5); the first two axes explained 93.4% of the total variation among the N-cycling bacterial communities. We found that the samples from CR soils were different from those from the other soils on the first axis. On the second axis, the samples from GR and OR were separated. In general, the TN, NH_4_^+^-N, TC, C:N ratio, and nitrate reductase activity (NRA) were significantly and positively associated with GR, while urease activity (UEA), NO_3_^−^-N, AK, and AOB were significantly and positively related to CR. The pH, EC and AN were significantly and positively related to OR. Nitrogen-fixing bacteria were positively associated with NH_4_^+^-N, TC and C:N, nitrifying bacteria were positively related to NO_3_^−^-N, AK and AP and denitrifying bacteria were positively associated with NH_4_^+^-N, TC, TN and C:N (Table 4).

## 3. Materials and Methods

### 3.1. Experimental Site

The experimental site was established in 2009 at the Qingpu Modern Agricultural Park of Qingpu, Shanghai (121.12°E, 31.15°N). The site of the rational rice cultivation patterns is located in the Yangtze River Delta. Rice is the dominant crop in this region and is cultivated once a year. The climate is a subtropical monsoon climate with a mean annual air temperature of 15.5 ℃ and a mean annual precipitation of 1200 mm. The average annual sunshine is 1960.7 h and the average soil pH is approximately 6.8. Before transplantation, the soil pH, electrical conductivity (EC) and available N, phosphorus and potassium levels at the site were determined to be 6.9, 0.13 mS cm^−1^, 1.70 g kg^−1^, 0.38 g kg^−1^, and 0.55 g kg^−1^, respectively. A rice-wheat rotation is the typical cropping system in this area. Rice seedlings were transplanted into the field in June and harvested in November. The growth of rice was classified into seven stages: Regreening, tillering, jointing, booting, heading, filling and maturing.

### 3.2. Experimental Design

Three rice cultivation patterns were established in this experiment:

(1) Conventionally cultivated rice (CR): A cultivation system with 100% chemical fertilizer;

(2) Green rice-frog cultivation (GR): A rice-frog cultivation system with a mixed 50% chemical and 50% organic fertilizer application;

(3) Organic rice-frog cultivation (OR): A rice-frog cultivation system with 100% organic fertilizer application. 

In both GR and OR fields, Chinese milk vetch (*Astragalus sinicus* L.) was seeded in the fields after the rice harvest and ploughed into the soil the following May as a basal fertilizer. The nitrogen fertilization was applied for each treatment with the same amount of 300 kg N ha^−1^ [32,33,34]. Tiger frogs (*Rana tigrina rugulosa*), which are highly adaptable to this environment, were introduced by the Zizaiyuan Agricultural Development Co. Ltd., Shanghai. At 15 days after rice transplanting, frogs large enough (≥20 g) to prey on pests were introduced into the paddy fields on sunny mornings. The fields maintained a feeding layer that was 3–5 cm deep during the rice growing season and were allowed to dry at the yellow ripening stage. At each site, a randomized complete block experimental design was used with three replicates. The timeline and major farming management practices are shown in Table 5. The timeline, dates of fertilization and major farming management practices are listed in Table 6.

### 3.3. Sampling and Measurements

#### 3.3.1. Soil

Soil samples were collected at eight intervals: Pre-transplantation, regreening, tillering, jointing, booting, heading, filling and maturation. Pre-transplantation soil samples were collected from the plough layer (0–20 cm) for each of the three replicates per treatment. At the other stages, five rice plants (with roots) were randomly selected from each plot, collected by an investigator wearing disposable gloves and then pooled. All rhizosphere soil was gently scraped from the roots and the soil was placed into sterile sealable plastic bags and transported back to the laboratory in an ice box containing liquid N. Each soil sample was separated into two parts. One portion was freeze-dried and stored at −80 °C for DNA extraction. One portion was air-dried, ground and passed through a 2-mm sieve to obtain a powder for analysis of the soil physicochemical characteristics: Soil pH, electrical conductivity (EC), available phosphorus (AP), available potassium (AK), available N (AN), total N (TN), total carbon (TC) and enzyme activity analyses. Total N and carbon were quantified by elemental analysis and isotope mass spectrometry (Agilent, CA, USA). Soil urease and protease activities were measured by the colorimetric method [35]. Urease activity was expressed as mg of NH_3_ produced g^−1^ oven-dried soil after 24 h of incubation at 37 °C. Protease activity was expressed as mg of tyrosine produced g^−1^ in oven-dried soil after incubation for 2 h at 40 °C. The activities of reductase and nitrite reductase were measured by benzene monosulfonic acid-acetic acid-α-naphthalene colorimetric method [36]. A unit of nitrate reductase activity was expressed as μmol of NO_2_^−^ produced by the reduction reaction of 1 g of soil in 24 hours at 30 °C; a unit of nitrite reductase activity was expressed as μmol of NO_2_^−^ reduced by 1 g of soil in 24 hours at 30 °C.

#### 3.3.2. Plant Harvesting and Grain Analysis

Uniform plants from each sub-plot were sampled for the determination of the straw and grain yield. The rice grain and straw were dried to a constant weight at 70 °C after harvest to determine the plant growth, N content and grain protein content. Total N was quantified by elemental analysis and isotope mass spectrometry (Agilent, CA, USA).

### 3.4. DNA Extraction

Rhizosphere soil samples were collected at the eight time points: Pre-transplantation, regreening, tillering, jointing, booting, heading, filling and maturation. DNA was extracted from 0.2 g of freeze-dried soil using a E.Z.N.A.^®^ soil DNA Kit (Omega Bio-tek, Norcross, GA, USA), following the manufacturer’s protocol. The DNA quality was evaluated by 1% sodium boric acid agarose gel electrophoresis. DNA quality and concentrations were determined using a NanoDrop 2000 spectrophotometer (Thermo Fisher Scientific Inc., Wilmington, DE, USA), and DNA was then stored at −20 °C prior to amplification.

### 3.5. Quantitative PCR

#### 3.5.1. PCR Amplification of Target Genes

The abundances of N-cycling genes (AOA, AOB, *nif*H, *nir*S, *nir*K and *nos*Z) were determined by quantitative real-time PCR (qPCR) on a CFX96 optical real-time detection system (Bio-Rad, Laboratories Inc., Hercules, CA, USA). Each 20 μL reaction contained 10 μL of SYBR Green (2X) PCR Master Mix (Life Technologies Corp., Carlsbad, CA, USA), 250 ng μL^−1^ bovine serum albumin (BSA), 0.2 μL of the forward and reverse PCR primers (both 20 μM), 1 μL of DNA template (containing 10–20 ng of total DNA) and 8.6 μL of double-distilled water (ddH_2_O). The primer sequences and annealing temperatures used in qPCR are listed in Table 7. Plasmid DNA containing fragments of *nif*H, AOA, AOB, *nir*K, *nir*S, or *nos*Z genes were used as qPCR standards. The specificity of the qPCR procedure was determined by melting curve analysis and agarose gel electrophoresis.

#### 3.5.2. Standard Plasmid and Standard Curve

We used the pEASY^®^-T1 Simple Cloning Kit (TransGen Biotech Co., Ltd.) to clone the purified target AOA, AOB, *nif*H, *nir*S, *nir*K and *nos*Z fragments. Three positive clones were sequenced. Standard plasmid DNA was extracted, adjusted to 10 ng μL^−1^, diluted seven times and amplified to establish the PCR efficiency. A ten-fold serial dilution of the plasmid DNA was subjected to a quantitative PCR assay in triplicate to generate a standard curve and to verify the amplification efficiency. The standard curve for AOA was y = −3.462x + 39.351 (R^2^ = 0.992); AOB, y = −3.597x + 39.908; *nif*H, y = −3.757x + 42.383 (R^2^ = 0.999); *nir*S, y = −3.829x + 41.078 (R^2^ = 0.990); *nir*K, y = −3.680x + 40.312 (R^2^ = 0.997); *nos*Z, y = −3.921x + 36.112 (R^2^ = 0.997). The amplification efficiencies ranged from 74.7 to 94.5%. The sequences of the AOA, AOB, *nif*H, *nir*K, *nir*S and *nos*Z gene clones were deposited in the GenBank database under accession numbers MH217678 to MH217683.

### 3.6. MiSeq Sequencing of 16S rRNA and Bioinformatics Analysis

DNA extracted from soil at the rice maturation stage was chosen for MiSeq sequencing. The amplification of 16S rRNA V4-V5 gene fragments was carried out using the primers 515F/907R (F: 5’-GTGCCAGCMGCCGCGG-3’ and R: 5’-CCGTCAATTCMTTTRAGTTT-3’) [42]. Amplicon library preparation and Illumina® MiSeq sequencing (Illumina, San Diego, USA) was performed by the Majorbio Bio-pharm Technology Co., Ltd. (Shanghai, China). Pairs of reads from the raw data were firstly merged with FLASH version 1.2.7, in which forward and reverse reads had an overlapping base length of >10 bp and base mismatches were not allowed [43]. Sequencing reads were processed with mothur version 1.31.1 [44]. The sequencing data were processed using the quantitative insights into microbial ecology (QIIME) [45]. Only sequences >200 bp in length with an average quality score >20 without ambiguous calls were included in subsequent analysis. After the sequences of the samples were sorted according to their barcodes, the barcode and primer sequences were deleted. A total of 298,045 high-quality 16S rRNA reads were obtained, and sequences of the 16S rRNA gene were binned into operational taxonomic units (OTUs) at the 97% similarity level. The raw sequencing reads were submitted to the National Center for Biotechnology Information Short Read Archive under accession numbers PRJNA471035 and SAMN09197674.

### 3.7. Statistical Analysis

For each variable measured in soil, the results were subjected to an analysis of variance (ANOVA), followed by Turkey test (α = 0.05) to determine the differences between the individual treatments (SPSS 20, IBM Corp., Armonk, NY, USA). Images were generated using Origin 8.0 (OriginLab Co., MA, USA). The Chao 1, abundance-based coverage estimator (ACE), Shannon, and InvSimpson indexes were calculated to estimate the α-diversity of each sample using mothur. We used the R package “DESeq2” to calculate the differential abundances of OTUs (i.e., the log2-fold change in the relative abundance of each OTU) as the control and used the “ggplot 2” package to plot volcano plots. Vegan heat maps were generated to show differences in the community compositions based on the dominant OTUs (with a relative abundance >1%) with the “pheatmap” package in R (a software environment for statistical computing and graphics). A Venn diagram was generated using the “Vennerable” package in R (MathSoft, Needham, MA, USA). Clustering was carried out using the “complete linkage” method, and a heatmap based on clustering was generated with the package “hclust” in R. Redundancy analysis (RDA) was performed to summarize the variations in N-cycling communities that could potentially be explained by the variables quantified (treatments, all physicochemical parameters) using the Canoco 4.5 software (Microcomputer Power, Ithaca, NY, USA).

## 4. Discussion

### 4.1. Different Rice Cultivation Patterns Drive Soil Enzymes Change

Soil enzymes are highly catalytic proteins released from the decomposition of soil microorganisms, plant root exudates, animals and plant residues. These enzymes directly participate in the process of matter and energy transformation in soil and are considered to be an important part of the soil ecology [46,47,48]. The soil urease activity can affect the contents of available N in the soil, and the NH_4_^+^ produced by the hydrolysis of urea is one of the main sources of plant N [49]. This study found that the urease activity of CR was the highest because a large amount of urea was used in the conventional cultivation of rice. Proteases are involved in the conversion of amino acids, proteins, and other N-containing organic compounds in soils [50]. In the GR and OR fields, organic fertilizer was applied to the rice field which stimulates microorganisms to produce proteases. The microbial hydrolysates were one of the N sources for rice with the application of a high amount of green and organic manure in the GR and OR paddy fields. Nitrate reductase and nitrite reductase are two key denitrifying enzymes involved in denitrification in soil, and their activities reflects the strength of denitrification in soil [51]. Under anaerobic conditions, nitrate reductase catalyzes the reduction of nitrate to nitrite, NO_2_^−^ is reduced to NO by nitrite reductase and NO is reduced to the greenhouse gas N_2_O. Our study indicated that the nitrate reductase activity in GR soil was significantly higher than that in OR and CR soil and there were no significant differences in nitrite reductase activity among the three patterns. The reason for this result may be that the bacteria-related nitrate reductase activity increased with the increasing application of N and carbon sources. The application of nitrogen fertilizer can increase the activity of denitrifying bacteria in soil, organic manure and easily decomposed organic matter such as glucose that can greatly stimulate the activity of denitrifying bacteria in soil, converting nitric acid to gas [52,53]. It is concluded that rice-frog cultivation methods (GR and OR) significantly increased soil protease and nitrate reductase activities but decreased urease activity.

### 4.2. Soil N-Cycling Genes of Different Rice Cultivation Patterns 

The N-cycling gene copy numbers increased after transplantation and subsequently decreased until the filling stage. The abundance of rhizosphere microbes had a correlation with the developmental stage of the rice plant [48]. When rice plants from all genotypes were well established in the vegetative phase of growth, the rhizosphere-associated microbial communities were very active at the jointing stage [46,47]. Biological nitrogen fixation can transform N_2_ to the biologically usable NH_4_^+^-N, which is also generated through soil mineralization [54]. This process is essential to catalyze nitrogen fixation, and the *nif*H gene is often used as a marker gene for the molecular analysis of nitrogen-fixing microbes [55]. In our study there were no significant differences in *nif*H abundance among the three rice cultivation patterns, which was similar to previous research in general [56,57]. In 2012, Fierer et al. found that N fertilizer application significantly increased the copies of soil function genes [58]. Anammox microbes use NO_2_^−^ as an electron acceptor to convert NH_4_^+^ into N_2_, and this NO_2_^−^ is provided by AOA or AOB, which convert NH_3_ into NO_2_^−^. Some previous studies have shown that the analysis of the *amo*A gene indicated a greater abundance of AOA than AOB in marine environments, undisturbed soils and some agricultural soils [37,59]. Nevertheless, some scholars have found that AOB accelerates the process of NH_4_^+^ oxidation in N-rich soils [60]. It was revealed that AOB are favored by inputs of urea and high concentrations of urease activity, which corresponds to high rice yields [61]. The ratio of AOA to AOB may serve as a new biological indicator for wetland assessment and management [26]. We found the ratios of AOA to AOB of CR, GR and OR, which were 0.08, 2.21 and 6.79, respectively. The differences among them were significant, where the rice-frog cultivation paddy ecosystem could improve paddy health conditions. Denitrification, the full or partial dissimilative reduction of NO_3_^−^ by microbes to N_2_, is the primary pathway producing N_2_O emissions from soil. Nitrite is converted to NO by nitrite reductase (NIR), the most widely used markers for which are *nir*K and *nir*S. The *nir*S, *nir*K and *nos*Z abundances increase when the total N and NH_4_^+^ concentrations are raised through external inputs, as long as the moisture, pH and organic C levels are favorable for denitrification to occur [62]. Our study indicated that there were no significant differences in the *nir*S and *nos*Z gene copy numbers between CR and OR, which were significantly higher than those in GR. Previous studies showed that the *nir*K, *nir*S and *nos*Z genes were favored by inputs of urea and high concentrations of N urease activity [63]. This indicated that the abundance of denitrifying bacteria in paddy soil responded significantly to nitrogen fertilizer, but their community structure was relatively stable. We believed that N can directly or indirectly affect the survival mechanism of soil microbes, and N addition makes the soil more conducive to copiotrophic microbial community growth. From the results of this study, denitrifying bacteria containing *nir*K were more sensitive to fertilization, however the community of denitrifying bacteria containing *nir*S and *nos*Z changed slightly and was relatively stable under long-term fertilization.

### 4.3. Microbial Community Structure Change among Different Rice Cultivation Patterns

As the microbial community structure is usually related to the soil properties in the rhizosphere, we further investigated the bacterial community composition of millet rhizosphere soil for the presence of specific bacteria indicative of different soil properties. The bacterial α-diversity was affected by GR and OR. The Chao1 and ACE indexes indicated that the OR pattern significantly increased the bacterial abundance compared to the CR pattern. Similar results were also found in previous studies in arable soil [64]. Urukawa and Bernhard indicated in 2017 that moderate conditions may increase interactions among microbes, eukaryotic microbes and macro-organisms, and that extreme conditions decrease species abundance but increase cell size and biomass, indicating a potential use of microorganisms as effective biological indicators for wetland management [27]. The paddy conditions of GR and OR were more moderate than that of CR. Bacterial communities across all treatments were dominated by the phyla *Proteobacteria*, *Acidobacteria*, and *Chloroflexi*, which is in agreement with a previous study based in paddy rhizosphere soils [25]. In nearly all freshwater wetlands, *Proteobacteria* dominated microbial communities at the phylum level [65]. In our study, the CR and GR soil had a significantly greater proportion of *Proteobacteria* and *Spirochaetae* than the OR soil, whereas *Chloroflexi* was mostly depleted in the GR soil. The OR soil had a significantly greater proportion of *Gemmatimonadetes*, *Planctomycetes* and *Chlorobi* than the CR or GR soil. Bacterial growth is often limited by C availability, even in soils with a high C:N ratio [66]. Microbial communities involved in different biogeochemical processes of paddy fields shift markedly. Various environmental parameters such as nutrient level, pH and water level may be changed and these changes should be quickly reflected by microbial communities [26]. Fast-growing copiotrophic bacteria proliferate soon after readily available C substrates are applied to the soil and decreased later, and the growth of slow-growing oligotrophic bacteria recovers as substrate C availability declines over time. As anticipated, the bacterial communities in GR soil were similar to those in OR soil. There is a possibility that allochthonous inputs to the bacterial community from organic amendments contributed to the alteration in the soil bacterial community composition. Recycling livestock manure in agro-ecosystems to partially substitute synthetic fertilizer N improves food production, reduces various N losses and increases SOC storage [67]. The microbes in manure that are well adapted to the gut environment are less competitive than indigenous microbes in soils. The soil microbial community composition and population not only are a simple result of the microorganisms in soils but also may be an outcome from specific environmental pressures. The application of organic fertilizer improves the ability of microorganisms to use soil available nitrogen, causing more soil available nitrogen to be assimilated into the transient storage reservoir of soil organic nitrogen and then remineralized into plant available nitrogen. The relationship between frog activity and soil microorganisms requires further verification. Several key environmental factors could explain the variation in microbial diversity [30]. It is well recognized that soil pH is a dominant factor and regulates bacterial community composition and diversity [68]. Our results showed that OR could increase bacterial community richness and diversity. *Proteobacteria*, *Acidobacteria* and *Chloroflexi* were the most dominant phyla. The paddy conditions of GR and OR were more moderate than that of CR. The relative abundance of *Proteobacteria* in CR and GR was higher than that of CR, meaning that the amount of nutrients in GR and OR was high.

### 4.4. N-Cycling Bacterial Community Abundance Was Effected by Rice Cultivation Pattern 

Under field conditions, even when completely flooded, due to the oxygenation of rice roots, there is strong nitrification on the root surface and rhizosphere of rice. The resulting NO_3_^−^-N plays an important role in the nitrogen nutrition of rice. Nitrification is a key process driven by microorganisms, which converts reduced nitrogen (N) from ammonium (NH_4_^+^) to nitrate (NO_3_^−^) via nitrite (NO_2_^−^) [69]. Thus, the suppression of nitrification and the maintenance of N fertilizer in the reduced form are critical steps to increase fertilizer-N retention in soils and to improve the N-use efficiency (NUE) of crops with a purpose of enhancing agricultural production and environmental protection. The importance of the nitrification in paddy fields has been mostly neglected because the popular view is that nitrification is an aerobic process, whereas paddy fields are in a flooded and consequently anaerobic environment [70]. However, nitrification still occurs in aerobic microenvironments, such as the soil-water interface and the rice rhizosphere. Recently, Yang et al. indicated that nitrifying microorganisms may be better adapted than so far thought to low oxygen environments, such as paddy soils during the flooding period, enabling the possibility that nitrification may occur extensively in paddy soils [71]. A recently isolated new species of AOB, *Nitrosospira* lacus, showed a high level of adaptation to rice fields where *Nitrosococcus* instead is normally found in marine environments [72]. At present, ammonia-oxidizing bacteria can be divided into three genera, *Nitrosomonas*, *Nitrosospira* and *Nitrosococcus*, which were all detected in our study. In the CR field, a large amount of urea was applied, which was hydrolyzed to ammonium carbonate or ammonium bicarbonate through soil urease action. The relative abundance of nitrifying bacteria was significantly different in the CR, GR and OR soils, and the relative abundance in CR soil was significantly higher than that in GR soil, which was higher than that in OR soil. There were findings that indicated that the abundance of *Nitrosomonas* in wetland nutrients is high [27]. The soil of CR was more nutrient-rich than GR or OR.

Denitrifying microorganisms are prevalent in soil, accounting for approximately between 0.5 and 5% of the total bacterial population. The long-term application of chemical fertilizers will lead to changes in the community structure of denitrifying bacteria in soil. The pH of the soil was not significantly affected in any of the three methods. Therefore, the pH was not the main factor that was significantly altering the denitrifying community’s diversity in this study. The research showed that the soil C:N ratio, TC, TN and NH_4_^+^-N were the most dominant environmental factors influencing the N-cycling community. Fertilization is one of the important factors that increases the soil microbial biomass and the denitrification rate [65]. The carbon and nitrogen sources in soil significantly affect denitrification. The higher the C:N ratio is, the stronger the denitrification rate [73]. Both *Thiobacillus* and *Rhizobium* belong to *Proteobacteria*, which was the most dominant bacterial phylum. Rice-frog cultivations significantly increased the relative abundance of denitrifying bacteria, but decreased the relative abundance of nitrifying bacteria.

## 5. Conclusions

Rice-frog cultivation significantly increase soil protease, nitrate reductase activities, the *nir*S gene copy number and the relative abundance of denitrifying bacteria but significantly decrease urease activity and the relative abundance of nitrifying bacteria. The bacterial community abundance and diversity of OR soil was significantly higher than that of GR or CR soil. The cumulative relative abundances of nitrogen-cycling bacteria, the plant N content, the grain protein content and the plant dry matter quality with GR was significantly higher than that of CR or OR and nitrogen use efficiency (NUE) highest in GR. *Thiobacillus* and *Rhizobium* were the dominant nitrogen-fixing and denitrifying bacteria and *Nitrospira* and *Nitrosomonas* were the dominant nitrifying bacteria. We observed that the N-cycling bacterial community was positively correlated with the TC, TN, and C:N ratio.

## Figures and Tables

**Table 1:** Grain protein content, plant N content and rice yield with each pattern.

Pattern	Grain Protein Content (%)	Plant N Content (%)	Dry Matter Biomass (g plant^−1^)	Rice Yield (kg ha^−1^)
CR	7.44 ± 0.06 b	1.05 ± 0.01 b	84.56 ± 3.23 a	8827.56 a
GR	9.72 ± 0.42 a	1.35 ± 0.02 a	86.95 ± 4.12 a	8650.38 a
OR	7.94 ± 0.10 b	0.97 ± 0.02 b	74.14 ± 5.02 b	7350.69 b

CR: Conventionally cultivated rice; GR: Green rice-frog cultivation; OR: Organic rice-frog cultivation. Different letters within columns indicate significant differences at *p* < 0.05.

**Table 2:** Correlation analysis for soil enzyme activities, N-cycling gene copy numbers and soil variables at the whole rice stage.

	UEA	PTA	AOA	AOB	*nir*K	*nif*H	*nir*S	Plant N
URA	-	-	-	-	-	-	-	-
PRO	-	-	-	-	-	-	-	-
NRA	-	-	-	-	-	-	-	-
NiRA	-	0.52 *	-	-	-	-	-	-
AOA	0.61 **	-	-	-	-	-	-	-
AOB	0.66 **	-	0.68 **	-	-	-	-	-
*nir*K	0.81 **	-	0.85 **	0.85 **	-	-	-	-
*nif*H	0.61 **	-	0.72 **	0.67 **	0.78 **	-	-	-
*nir*S	0.62 **	-	0.47 *	-	0.45 *	0.53 *	-	-
*nos*Z	0.68 **	0.44 *	0.77 **	0.53 *	0.70 **	0.53 *	0.78 **	-
Plant N	0.84 **	-	-	-	-	-	-	-
Grain Pro	−0.73 *	-	-	-	-	-	-	0.94 **
Rice yield	-	−0.67 *	-	0.83 **	-	-	-	-

Single asterisks (*) represent significantly correlation at *p* < 0.05 (*n* = 21); double asterisks (**) represent significant correlation at *p* < 0.01 (*n* = 21); ns means non-significant. Seven stages of the rice season, including regreening, tillering, jointing, booting, heading, filling and the maturing period were analyzed through Pearson correlation analysis. UEA: Urease activity; PTA: Protease activity; NRA: Nitrate reductase activity; NiRA: Nitrite reductase activity; Grain Pro: Protein content of grains.

**Table 3:** α-diversity indexes of bacteria in the different patterns.

Index	Chao 1	ACE	Shannon	InvSimpson
CR	2391.55 ± 17.52 b	2396.35 ± 25.90 b	6.58 ± 0.03 b	293.07 ± 5.38 c
GR	2398.68 ± 60.50 b	2397.42 ± 77.19 b	6.61 ± 0.02 b	326.91 ± 2.52 b
OR	2654.24 ± 27.03 a	2646.10 ± 30.40 a	6.81 ± 0.03 a	406.75 ± 18.23 a

Different letters indicate significant differences among the fertilization treatments by one-way ANOVA (Tukey, *p* < 0.05). CR: Conventionally cultivated rice; GR: Green rice-frog cultivation; OR: Organic rice-frog cultivation; ACE: Abundance-based coverage estimator.

**Table 4:** Correlation analysis for N-cycling bacteria and soil variables at the maturing stage.

	NO_3_^−^-N	NH_4_^+^-N	PH	EC	TC	TN	C:N	AK	AP	AN	N1
NO_3_^−^-N											
NH_4_^+^-N	−0.88 **										
PH	−0.84 **										
TC		0.96 **	0.90 **								
TN		0.70 *			0.67 *						
C: N		0.84 **			0.91 **						
AK	0.95 **		−0.92 **	−0.88 **							
AP	0.73 *		−0.93 **	−0.91 **				0.77 *			
AHN	−0.91 **		0.98 **	0.93 **				−0.92 **	−0.94 **		
N1	−0.76 *	0.92**			0.94 **		0.86 **	−0.70 *			
N2	0.72 *		−0.80 *	−0.81 **				0.75 *	0.74 *	−0.75 *	
N3		0.89 **			0.95 **	0.74 *	0.80 **				0.87 **

Single asterisks (*) represent significant correlations at *p* < 0.05 (*n* = 9); double asterisks (**) represent significant correlations at *p* < 0.01 (*n* = 9). Significant correlation (*p* < 0.05, *n* = 9) are shown in this table. The soil variables included the electrical conductivity (EC), total carbon (TC), total nitrogen (TN), Carbon:Nitrogen ratio (C:N), available potassium (AK), available nitrogen (AN), and available phosphorus (AP). N1: Nitrogen-fixing bacteria, N2: Nitrifying bacteria, N3: Denitrifying bacteria.

**Table 5:** N fertilization scheme for each treatment.

Patterns	Pre-transplanting			Jointing Stage		Heading Stage
	Chinese milk vetch	rapeseed cake	bulk blending fertilizer	bio-organic fertilizer	bulk blending fertilizer	urea
CR	-	-	150	-	75	75
GR	22.5	127.5		-	75	75
OR	22.5	127.5	-	150	-	-

The unit for the fertilizer application rate was kg·N·ha^−1^. The urea (total N content, 46%) was obtained from the Luxi Chemical Group Co., Ltd. (Shanghai, China). The bulk blending fertilizer (total N content, 26%) was obtained from the Shanghai Changzheng Fertilizer Technology Co., Ltd. (Yangzhou, China). The bio-organic fertilizer (total N content, 6.2%) was obtained from the Nanjing Lvchuang Ecological Technology Co. Ltd. (Nanjing, China). The rapeseed cake (total N content, 5.3%) was obtained from the Shanghai Xinhelv Bio-organic Fertilizer Co. Ltd. (Shanghai, China). CR: Conventionally cultivated rice; GR: Green rice-frog cultivation; OR: Organic rice-frog cultivation.

**Table 6:** Timeline and major farming management practices.

Rice Stage	Day	Dates	Major Farming Management Practices
Pre-transplanting	31	14 May–14 Jun.	Ploughed Chinese milk vetch in GR and OR (May 14), applied 100% rapeseed cake in GR and OR, 67% bulk blending fertilizer in CR (Jun. 14).
Regreening	15	15 Jun.–30 Jun.	
Tillering	21	1 Jul.–22 Jul.	Put tiger frogs into fields (6000 frogs ha^−1^, Jul. 1). Applied 33% bulk blending fertilizer in CR and 100% in GR, 100% bio-organic fertilizer in OR (Jul. 15).
Jointing	20	23 Jul.–12 Aug	
Booting	18	13 Aug.–31 Aug.	Applied 100% urea in CR and GR (Aug. 15).
Heading	17	1 Sep.–18 Sep.	
Filling	20	19 Sep.–9 Oct.	
Maturing	19	10 Oct.–29 Oct.	Sowed Chinese milk vetch in GR and OR (Oct. 20)
Harvesting	16	30 Oct.–14 Nov.	Harvested on Nov. 14.

The specific fertilizing of urea, bulk blending fertilizer, bio-organic fertilizer, rapeseed cake and Chinese milk vetch were calculated from their N contents of 46%, 26%, 6.2%, 5.6% and 0.5%, respectively. CR: Conventionally cultivated rice; GR: Green rice-frog cultivation; OR: Organic rice-frog cultivation. Tiger frogs were introduced by the Zizaiyuan Agricultural Development Co., Shanghai (Shanghai, China).

**Table 7:** Group-specific primers for the qPCR gene quantification assays.

Target Gene	Primer Set	Primer Sequence (5′-3′)	Reference
AOA ^a^ (*amo*A)	Arch-*amo*AF Arch-*amo*AR	STAATGGTCTGGCTTAGACG GCGGCCATCCATCTGTATGT	[37]
AOB ^b^ (*amo*A)	*amo*A1F *amo*A2R	GGGGTTTCTACTGGTGGT CCCCTCKGSAAAGCCTTCTTC	[38]
*nif*H	*nif*H-F *nif*H-R	AAAGGYGGWATCGGYAARTCCACCAC TTGTTSGCSGCRTACATSGCCATCAT	[39]
Cd-nitrite reductase (*nir*S)	Cd3aF R3cd	GTSAACGTSAAGGARACSGG GASTTCGGRTGSGTCTTGA	[40]
Cu-nitrite reductase (*nir*K)	FlaCu R3Cu	ATCATGGTSCTGCCGCG TTGGTGTTRGACTAGCTCCG	[40]
Nitrous oxide reductase (*nos*Z)	*nos*Z2F *nos*Z2R	CGCRACGGCAASAAGGTSMSSG CAKRTGCAKSGCRTGGCAGAA	[41]

^a^ Ammonia Oxidizing Archaea, ^b^ Ammonia Oxidizing Bacteria.
